# Human in vitro models for Fabry disease: new paths for unravelling disease mechanisms and therapies

**DOI:** 10.1186/s12967-024-05756-w

**Published:** 2024-10-24

**Authors:** Carla Borisch, Thomas Thum, Christian Bär, Jeannine Hoepfner

**Affiliations:** 1 Institute of Molecular and Translational Therapeutic Strategies (IMTTS), Hannover Medical School, Hannover, Germany; 2 Center for Translational Regenerative Medicine, Hannover Medical School, Hannover, Germany; 3 Fraunhofer Institute for Toxicology and Experimental Medicine ITEM, Hannover, Germany

## Abstract

Fabry disease is a multi-organ disease, caused by mutations in the *GLA* gene and leading to a progressive accumulation of glycosphingolipids due to enzymatic absence or malfunction of the encoded alpha-galactosidase A. Since pathomechanisms are not yet fully understood and available treatments are not efficient for all mutation types and tissues, further research is highly needed. This research involves many different model types, with significant effort towards the establishment of an in vivo model. However, these models did not replicate the variety of symptoms observed in patients. As an alternative strategy, patient-derived somatic cells as well as patient-independent cell lines were used to model specific aspects of the disease in vitro. Fabry disease patients present different phenotypes according to the mutation and the level of residual enzyme activity, pointing to the necessity of personalized disease modeling. With the advent of induced pluripotent stem cells, the derivation of a multitude of disease-affected cell types became possible, even in a patient-specific and mutation-specific manner. Only recently, three-dimensional Fabry disease models were established that even more closely resemble the native tissue of investigated organs and will bring research closer to the in vivo situation. This review provides an overview of human in vitro models and their achievements in unravelling the Fabry disease pathomechanism as well as in elucidating current and future treatment strategies.

## Introduction

Fabry disease is a disorder of the glycosphingolipid metabolism. It is a rare, X-linked disease first described [1] in 1898 as a variation of exanthema occurring in different densities and colors, and therefore also named *Angiokeratoma corporis diffusum* [2, 3]. The disease is caused by mutations in the *GLA* gene, located on the X-chromosome and encoding the enzyme α-galactosidase A (α-Gal A), which catalyzes the hydrolysis of globotriaosylceramide (Gb3) [4]. The deficiency of this enzyme leads to a progressive accumulation of Gb3 and other neutral glycosphingolipids inside the cells, which are the suspected cause for the manifold clinical manifestations that progress over time [5].

**Fig. 1:**
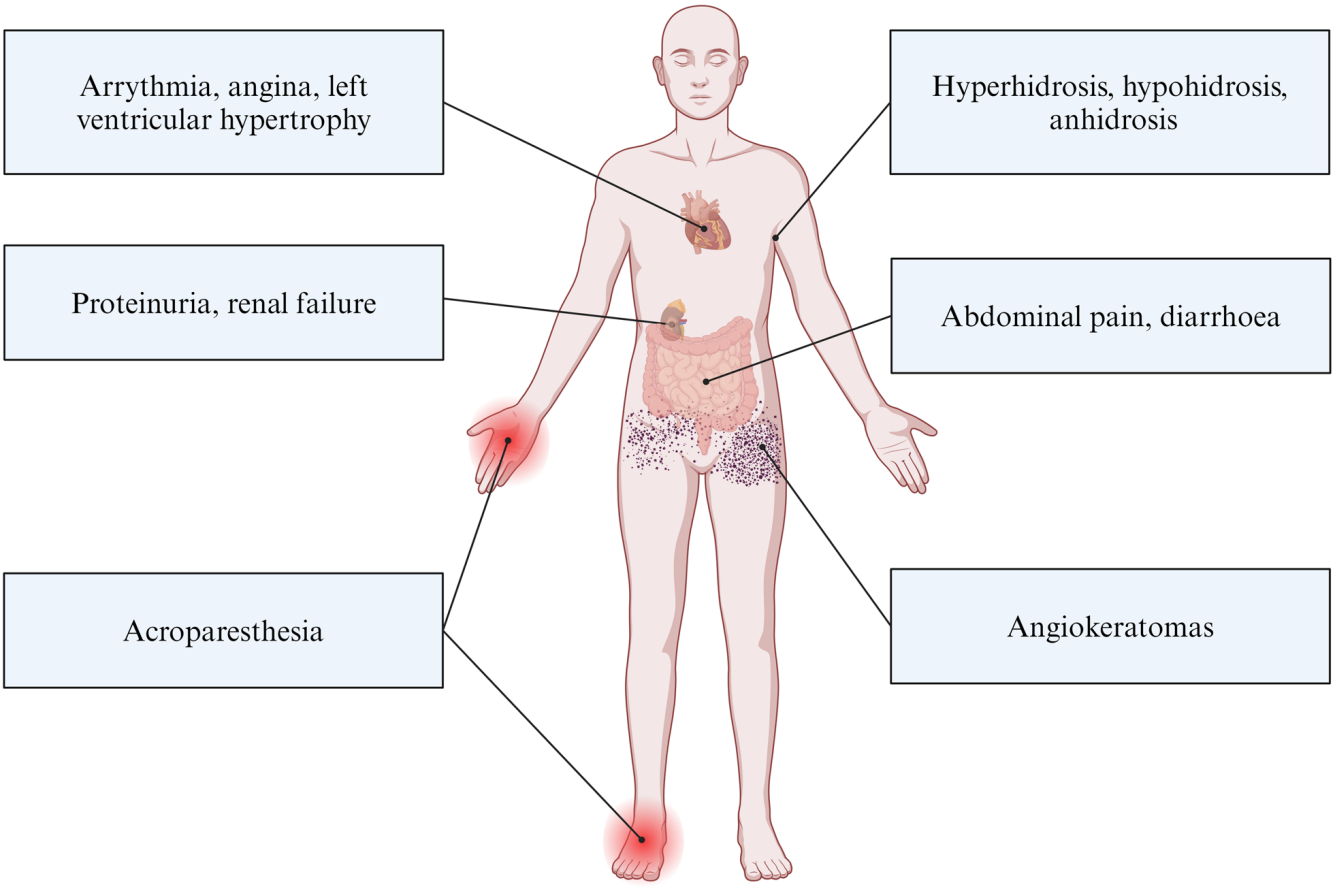
Main symptoms of Fabry disease

The accumulation of Gb3 in the lysosomes affects the whole body, and Fabry is considered a multi-systemic disease [6]. The main symptoms of Fabry disease are diverse and include angiokeratomas, sweating abnormalities (hyper- or hypohidrosis, anhidrosis), lymphedema [7], acroparesthesias, hearing loss, tinnitus, proteinuria, renal failure, arrhythmias, angina, left ventricular hypertrophy, strokes, abdominal pain and diarrhoea[5, 8] (Fig. 1).

The incidence of Fabry disease has been estimated to range between 1 in 40,000 and 1 in 117,000 births [5, 9], although the number varies depending on the geographical region assessed, and can be as high as 1 in 3,100 male births [10]. Mutations can vary from point mutations to rearrangements in any region of the *GLA* gene [4] and around 57% of mutations are missense [11]. Mutations can cause either a more severe phenotype with enzyme activity levels being less than 5% of the normal mean, referred to as classical phenotype or cause a milder phenotype with higher residual enzyme activity [12, 13]. In both cases, a single point mutation is sufficient to produce the phenotype [13] while for most mutations, there is no clear genotype–phenotype correlation [14].

Even if the disease affects the body as a whole, some organs are more affected than others. One of the main organs involved in worsening their life quality is the heart. The Gb3 accumulation in the cardiomyocytes is thought to contribute to the development of cardiac hypertrophy and the involvement of the conduction system contributes to the development of arrhythmias [4]. Whether these are direct or secondary effects is still not understood. Nevertheless, cardiac manifestations can arise at early age, with children clearly showing signs of cardiac involvement, such as left ventricular hypertrophy [15]. In severe cardiac hypertrophy, the wet weight of glycosphingolipid deposits can be as high as 2% of total heart weight [16]. This excessive storage is hypothesized to activate secondary cellular pathways that may lead to apoptosis, autophagy, necrosis and fibrosis [4].

Two different treatment options are currently available, and the earlier treatment is initiated, the lower is the risk of severe organ damage. Enzyme replacement therapy (ERT) was approved in Europe and the USA in 2001 and 2002, respectively [17]. The early treatment start can delay or mitigate the effects of the Gb3 accumulation, especially in the kidney and the heart [18]. After treatment, patients showed a reduction in the concentration of Gb3 in the plasma [19, 20], liver, skin, kidney and heart [21], along with a reduction of left ventricular hypertrophy [22]. Data show that long-term treatment can prolong survival and reduce the risk of serious clinical events [20]. It has also been suggested that ERT can influence on circulating miRNA levels and could help understand the disease pattern and which processes would be involved in improving the symptoms [23].

The second treatment option is a chaperone therapy with migalastat [24]. This treatment option has many beneficial effects that are similar to the enzyme replacement therapy, with reduction of Gb3 in the kidney and reduction of left ventricular hypertrophy [25]. Chaperone therapy could also be used in combination with enzyme replacement therapy [26]. However, it is restricted to certain amenable mutations and is therefore only available to a subset of patients.

Further treatment options are currently under development including substrate reduction, gene and mRNA therapies. mRNA therapy has been shown to increase the levels of human α-Gal A in heart and kidneys of healthy and Fabry mice using LNPs as a delivery system [27]. This therapy uses pharmacological intervention to inhibit the biosynthesis of the substrate that will cause the accumulation. In Fabry disease, lucerastat, a glucosylceramide synthase inhibitor, has been studied for its efficacy [28] (NCT02228460) in reducing the precursors of Gb3 [29]. The development of gene therapies with the aim of targeting cells that could overexpress α-Gal A, and subsequently secrete it to peripheral organs [24] are ongoing. Lentivirus-mediated gene therapy in Fabry patients with classical phenotype has shown to increase circulating and intracellular α-Gal A activity [30]. Although there are promising novel therapeutic approaches, further research is needed to better understand the disease mechanisms, develop therapies that target difficult to reach tissues and create adjuvant therapies that address secondary cellular processes caused by the enzyme deficiency.

Research on Fabry disease involves various model types, with a significant focus on developing an in vivo model. One such model is the *GLA* knockout mouse model, which demonstrated no Gb3 accumulation in the heart spleen and lungs, but could identify zebra bodies in kidneys and liver [31]. *GLA-*KO mice have also been used for investigation of pain and angiogenesis in normoxia and hypoxia conditions [32]. Another mouse model involves expressing a human mutant α-Gal A in mice with a *GLA* knockout background, along with overexpressing a Gb3 synthase and serve as a model to study active-site-specific chaperon therapy [33]. These mice showed Gb3 accumulation in their kidneys, similar to the other model [34], but also showed a lower level of Gb3 accumulation in the heart, which could only be achieved by the overexpression of human α1,4-galactosyltransferase to increase the production of Gb3. This effort, albeit valid, still does not reflect the patient situation. A *GLA* knockout rat model showed a similar pattern of glycosphingolipid accumulation in serum [35]. These also developed neuropathic pain and showed accumulation of neutral glycosphingolipids in dorsal root ganglia [34], as well as in kidney cells and the heart of aged rats [36]. The rat model also presents alterations in skin and [32]anomalies in the sebaceous ducts. However, these rats did not develop left ventricular hypertrophy, but rather wall thinning and presented other limitations such as lack of vascular pathology.

These data show that in vivo models do not fully reflect the range of symptoms associated with Fabry disease, presumably owing to their relatively short lifespan compared to humans. Therefore, in vitro models that focus on specific cell types may help to address some of these gaps. The cultivation of cells in vitro offers many advantages, including less bureaucracy and greater flexibility than in vivo studies. It also allows for the direct study of human cells, which can help to address translational concerns. These models also enable the use of the CRISPR/Cas9 system to create isogenic controls for diseases that derive from a genetic mutation [37]. This review summarizes human in vitro models of Fabry disease that are based on patient-derived cells, patient-independent cell lines or on pluripotent stem cells, including three-dimensional disease models. This variety of models is crucial for validating new therapies and offers hope for individuals with Fabry disease. The studies in Fabry disease included in this review were identified using Pubmed, ScienceDirect or in the individual websites of the respective Journals between January 2023 and April 2024 by using the search terms “Fabry disease” “Anderson-Fabry disease”, “fabry disease model”, “fabry disease in vivo models” and “Fabry disease in vitro models” either alone or in combination.

### Disease models based on patient-derived cells

**Fig. 2:**
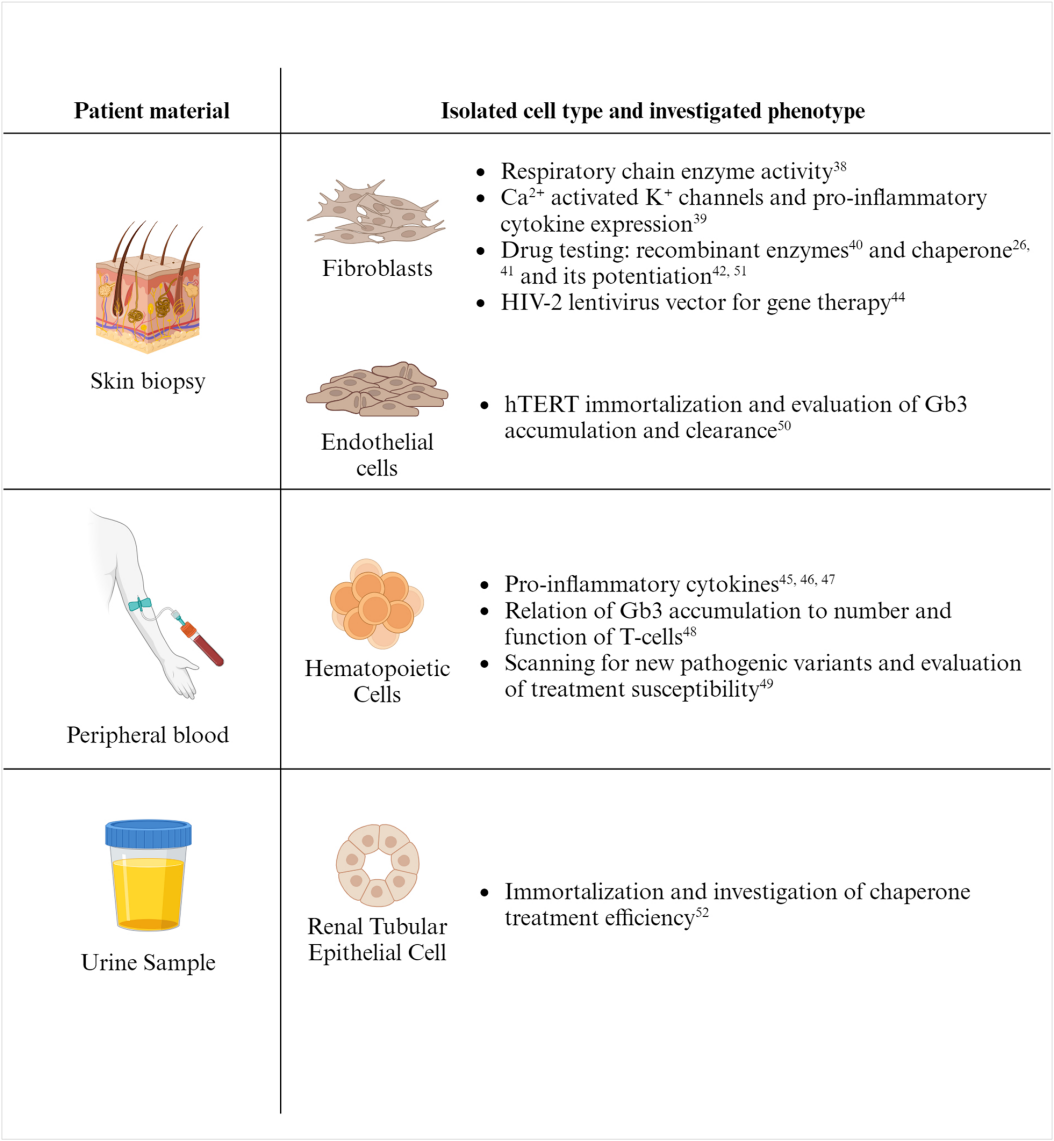
Overview of in vitro models derived from Fabry patients

Research using patient-derived cells has the significant advantage of preserving the patient-specific genotype and phenotype, which is of particular importance for investigating a disease with high genotype–phenotype variations such as Fabry disease. However, research with patient-derived cells is limited to those cell types that are easily accessible without major obstacles or ethical concerns for the patient and to cells that keep their unique characteristics during subsequent in vitro cultivation and experimentation. Currently, studies using patient-derived cells are limited to fibroblasts and endothelial cells derived from skin biopsies as well as to cells derived from body fluids such as hematopoietic cells and urine-derived cells (Fig. 2).

Studies using skin fibroblasts derived from such Fabry patient biopsies showed a reduced respiratory chain enzyme activity, along with a reduction of energy-rich phosphates AMP, ADP and CP, indicating an impaired mitochondrial energy supply and that this can play a role in the pathogenesis of Fabry disease through a proposed change in the properties of the mitochondria membrane [38], further implicating the mitochondrial dysfunction as part of the symptoms. Patient-derived fibroblasts were also used to study expression and electrical activity of Ca^2+^ activated K^+^ channels (K_Ca_1.1) and pro-inflammatory cytokine expression [39]. Patch-clamp analysis pointed to an impairment of K_Ca_1.1 upon Gb3 accumulation in fibroblasts and an activation of Notch signaling, finally resulting in an upregulation of pro-inflammatory cytokines, which could be a mechanism contributing to the cutaneous pain seen in patients. Moreover, cultured fibroblasts from Fabry disease patients offer an alternative to study pharmacological approaches. One study showed the efficient uptake of the recombinant enzymes agalsidase α and β and proved that these enzymes efficiently cleared out most of the Gb3 accumulations upon a 24 h in vitro treatment. In contrast, biopsy cultured tissues from patients receiving enzyme replacement therapy did not show such clearance in the same cell type [40]. The authors hypothesize that the enzyme uptake may be hindered by physical barriers in the tissue since cells that are of easier access to the enzyme, such as endothelial cells, have their deposits cleared. Such findings raise the concern of how to improve the enzyme delivery in patients. For the second approved Fabry disease therapy, the chaperone therapy, it was shown that administration of chaperones galactose and 1-deoxygalactonojirimycin (DGJ, migalastat) can help to improve the enzyme activity in cultured fibroblasts by stabilizing the mutant protein so it can be transported to the lysosome and execute its catalytic function [41]. Cultured fibroblasts also allowed studying the molecular interactions between potential chaperones and the α-Gal A protein, thereby opening more alternatives for improvement of the treatments for Fabry disease [26]. Unfortunately, such compounds can affect other steps of the glycosphingolipid metabolism, and therefore, new formulations are needed for a more efficient treatment [26]. In line with the chaperone therapy, the potentiation of chaperone activity was studied in cultured fibroblasts by evaluating the interaction between DGJ and acetylsalicylic acid and showing, in a dose dependent manner, an increased stabilization of α-Gal A upon acetylsalicylic acid supplementation [42]. Among the novel treatment options currently under investigation, lucerastat has been shown to decrease Gb3 levels in 15 different patient skin punches, with a median reduction of 77% and higher volume of distribution than ERT, making it available in tissues that are difficult to reach. This makes it a promising substrate reduction therapy for patients with all mutation types and further research is warranted [43]. In addition to the substrate reduction therapy, gene therapy approaches as a curative treatment option are under investigation. To this end, patient-derived fibroblast cultures were used to evaluate a HIV-2 lentivirus vector and were able to demonstrate efficient gene transfer and induction of functional enzyme activity, increasing the levels at least tenfold, along with Gb3 clearance upon lentiviral *GLA* transduction [44].

Disease modeling from patient derived cells is also possible with hematopoietic cells. Peripheral blood mononuclear cells (PBMCs) have been used in culture to evaluate their inflammatory state and the contribution of inflammation to the disease phenotype. Fabry PBMC cultivation revealed a pro-inflammatory state with increased production of interleukin-1β (IL-1β) and TNF-α caused by Gb3 accumulation, particularly in dendritic cells and monocytes [45]. Another study described a relationship between elevated cytokine expression (TNF-α and IL-1β) in male patient-derived PBMCs and pain, thereby making the connection between inflammatory triggers and Gb3 accumulation even more complex [46]. The authors could also show that simulation of a febrile state increase intracellular levels of Gb3 through the increase of TNF expression and secretion. Studying potential adjunctive treatments targeting inflammation, Fabry patient-derived PBMCs showed significantly reduced secretion of pro-inflammatory cytokines upon in vitro treatment with pentosan polysulfate (PPS), although the action mechanism is not clear [47]. Models of invariant natural killer T cells derived from patient PBMCs also could show that the accumulation of Gb3 negatively affected their numbers and cell function [48], impacting even further on the immune system. The use of hematopoietic cells in vitro is not limited to the research of immunological factors, patient-derived lymphocytes have also been employed in functional studies, identifying new pathogenic variants, and scanning for susceptibility of the variants to chaperone treatment in combination with in silico predictions of pathogenicity [49].

The cultivation time of primary cells is limited, which poses a significant challenge for modelling progressive and late-onset diseases such as Fabry disease. To overcome this hurdle, Shen et al. immortalized patient-derived endothelial cells by transduction with a retrovirus encoding for the human telomerase reverse transcriptase gene (hTERT). The culture time of resulting cells increased to up to 5 months and these cells proved superior disease modeling potential by means of presenting clear Gb3 accumulation and responding to α-Gal A treatment [50]. Also, Monticelli et al. used immortalized patient-derived cells, namely fibroblasts with different underlying *GLA* gene mutations, and showed that effective clearance of Gb3 by chaperone therapy could be strengthened by the use of curcumin, which mediated increase in α-Gal A activity and decrease in lysosomal markers possibly due to the disruption of the molecular function of Hsp90 [51]. Lenders et al. immortalized patient-derived urinary cells by retroviral introduction of the HPV type 16 E6 and E7 genes and, subsequently, investigated the in vitro amenability of certain mutations to chaperone therapy [52]. The results obtained from the patient-derived cells were validated by *GLA* knockout HEK cells overexpressing mutant *GLA,* demonstrating that such models are valuable for studying the disease in a patient-independent context.

### Disease models based on patient-independent cells

**Fig. 3:**
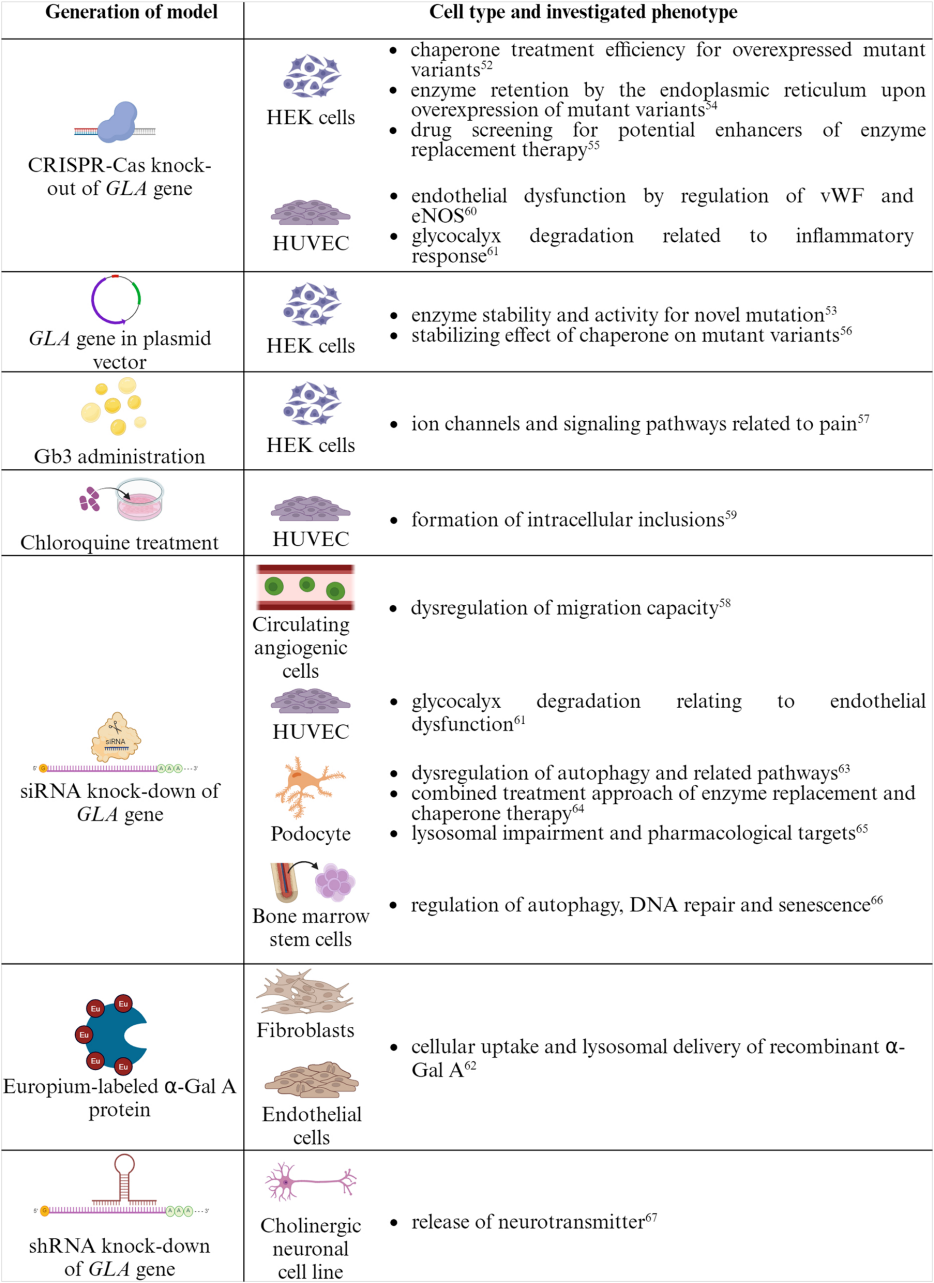
Summary of patient-independent models for Fabry disease

Despite the unique advantages of primary, patient-derived cells, these cell models have some important limitations. Not only the limited cultivation time previously described, but also the restriction to those cells that can easily be accessed from humans and readily cultivated confines the spectrum of target cells available for disease modeling. The latter is especially decisive for modeling a multisystemic disease like Fabry disease. Furthermore, though the conservation of the patient-specific phenotype in primary, patient-derived cells allows patient-specific disease modeling, an excessive variation between individual disease models cannot be neglected. For studies rather aiming to identify disease-specific mechanisms that apply to a huge variety of affected patients or to identify treatment options that show beneficial effects in a majority of patients, disease models that are independent from individual genetic backgrounds might be superior. A summary of in vitro models for Fabry disease that are independent from patient cells is depicted in Fig. 3.

In this regard, HEK cells represent a robust cell line with a broad range of applications, e.g. the characterization of individual mutations with their distinct enzyme activity. To this end, HEK cells were transfected with plasmids containing the *GLA* cDNA with a c.280 T > C mutation for a functional evaluation [53]. This mutation was identified from a Chinese family presenting nephropathy and specific conformational changes in the mutated enzyme involving a disulphide bridge were revealed that reduced its activity to 40% of the normal level [53]. HEK cells were further used for an assessment of enzyme stability and the involvement of specific intracellular compartments like the endoplasmic reticulum (ER) in pathological mechanisms of Fabry disease. A CRISPR/Cas9-mediated knockout of the *GLA* gene together with the overexpression of a number of different mutant variants allowed establishing a correlation between enzyme activity levels and ER retention [54] and, thereby, furthered the understanding of the full clinical presentation of Fabry disease. Studies including mutations already known in the clinics are excellent opportunities to understand the response that treatments could have and help choose a strategy. In a drug screening approach, *GLA* knockout HEK cells were treated with recombinant human α-Gal A protein and the proteasome inhibitor MG132 was identified as a potential enhancer for ERT by increasing the activity of the recombinant enzyme by twofold and clearing 30% more of the Gb3 deposits in patient derived fibroblasts [55]. Another approach to study enzyme stability, and especially susceptibility to chaperone treatment, used a urea-induced unfolding technique of mutant α-Gal A variants expressed in HEK cells, which is a valuable tool not only in the case of Fabry disease [56]. Moreover, in an attempt to ameliorate neuropathic pain of patients, HEK cells have been used to investigate the relationship between Gb3 accumulation and an acid sensing ion channel related to pain (ASIC1a). Gb3 accumulation resulted in activation of ASIC1a and, in turn, in activation of the pain associated ERK signaling pathway [57]. These findings point to a possible treatment of many patients presenting pain.

In addition to the insights into Fabry disease mechanisms obtained from HEK cell models, human umbilical vein endothelial cells (HUVECs) represent a meaningful tool for Fabry disease modeling since endothelial cells are known to be severely affected in Fabry disease [58]. In one study, immortalized HUVECs were treated with chloroquine, a lysosomal inhibitor, which reduced the activity of α-Gal A to a level of 20% of the control and resulted in the formation of intracellular inclusions typical for the Fabry disease phenotype [59]. The authors claimed that this cell model might allow investigations on secondary effects resulting from the observed inclusion bodies. Endothelial cells were also used to investigate the direct association between α-Gal A deficiency and endothelial dysfunction. In one study, disruption of the *GLA* gene increased the secretion of von Willebrand factor (vWF) up to fivefold, correlating with the decrease of endothelial nitric oxide synthase (eNOS) activity [60]. Thereby, this study suggested a direct regulation of vWF by α-Gal A through the activity of eNOS, which might be a potential mechanism of vascular dysfunction seen in patients. A lower expression of eNOS has also been suggested in untreated Fabry patients [58]. In another study, α-Gal A-deficient endothelial cells showed the degradation of the glycocalyx by heparinases because of an inflammatory response and the authors suggested that this might explain the endothelial dysfunction observed in Fabry patients [61]. The same study also clarified that the persistent degradation of the glycocalyx will result in a chronic inflammatory response in small vascular vessels and cause damage to the vascular endothelium. The use of conditioned media from circulating angiogenic cells from Fabry patients on healthy HUVECs was demonstrated to impair their tube formation capacity [58], which highlights the complexity of the symptoms in this disease.

With the aim of investigating treatment efficacy using in vitro models, a study using endothelial cells and fibroblasts revealed that the delivery of α-Gal A is different in each cell type [62]. In fibroblasts, the mannose-6-phosphate receptor pathway mediated uptake and delivery of α-Gal A to the lysosomes, whereas, at least in the applied in vitro system, the mannose-6-phosphate receptor pathway did not mediate lysosomal delivery of α-Gal A into endothelial cells. Therefore, the mechanisms leading to effective Gb3 clearance in endothelium observed in patients after enzyme replacement therapy [21] remain to be further elucidated.

Since Fabry disease patients are often affected from renal dysfunction and ultimately kidney failure, research on podocytes aimed to employ the underlying mechanisms. *GLA* knockdown podocytes were generated using RNAi techniques in combination with lentiviral gene transfer, and these cells showed the α-Gal A enzyme deficiency as well as Gb3 accumulation [63]. The authors could also show that the lack of enzyme activity and glycosphingolipid accumulation had functional consequences to the podocytes, such as dysregulated autophagy, as well as impairment of mTOR and AKT cascades, which may contribute to further cellular damage. In a follow-up study, the authors used a combination of recombinant α-Gal A and migalastat to clear the cells from Gb3 deposition. They could show a decrease in the accumulation of Gb3 after treatment [64]. However, a normalization of the dysregulated autophagy could not be observed and pro-fibrotic signaling was not reduced. They concluded a point of no return after which dysregulated cellular signaling does not reverse although Gb3 deposits were cleared from affected cells. Since podocyte injury extends beyond Gb3 accumulation, analysis of signaling networks in Fabry have shown the involvement of α-synuclein in lysosomal impairment and hints to pharmacological treatments modulating α-synuclein and the use of β_2_-adrenoreceptor agonists [65] as promising interventions.

Similar to the *GLA* knockdown in podocytes, *GLA* silencing by siRNA in bone marrow stem cells revealed alterations in cell biology and in cell cycle, including impaired autophagy and DNA repair which seems to be increasing the levels of senescence and apoptosis [66]. As it seems, autophagy and senescence are linked processes, although it is not always clear which one triggers the other, but in the case of mesenchymal stem cells and Fabry disease an impaired autophagic flux seems to be triggering the cell senescence [66], which can imbalance other cellular processes. An siRNA approach has also been applied to circulating angiogenic cells and showed that a 50% silencing of the *GLA* gene is enough to significantly impact their migration capacity [58]

To shed light on the mechanism by which α-Gal A deficiency leads to another commonly observed Fabry disease phenotype, namely neuronal dysfunction, Kaneski et al. applied a knockdown approach using a shRNA directed against *GLA* in a cholinergic neuronal cell line and observed reduced α-Gal A activity and pronounced Gb3 accumulation [67]. As a secondary effect, release of the neurotransmitter acetylcholine was reduced, and the authors concluded that this cell model might allow further investigations on neuronal functions in Fabry disease.

### Disease models based on pluripotent stem cells

The advent of the human iPSC technology opened many doors to the study of diseases. There was no longer the ethical concern of using embryonic stem cells or a need for frequent use of animal models, which is also an advantage when considering animal welfare and costs. These cells could now be used for understanding disease mechanisms, making drug screening easier and studying toxicology [68]. One advantage that the generation of pluripotent stem cells from somatic cells brings is the possibility to cultivate patient-derived cells and to study the consequences of the disease they may carry in different cell types in a patient-specific manner. Once in culture, such cell lines can be expanded and kept frozen, allowing experiments to continue without further patient involvement. Disease modelling with patient-derived iPSCs has further advantages such as the ability to obtain cell types that might otherwise be difficult to obtain [69]. As reviewed by Karagiannis et al*.* there are several different types of cells that can be differentiated from iPSCs, such as adipocytes, podocytes, renal tubular cells, cholangiocytes, hepatocyte-like cells, skeletal muscle cells, endothelial cells and also cardiac cells [69].

**Fig. 4:**
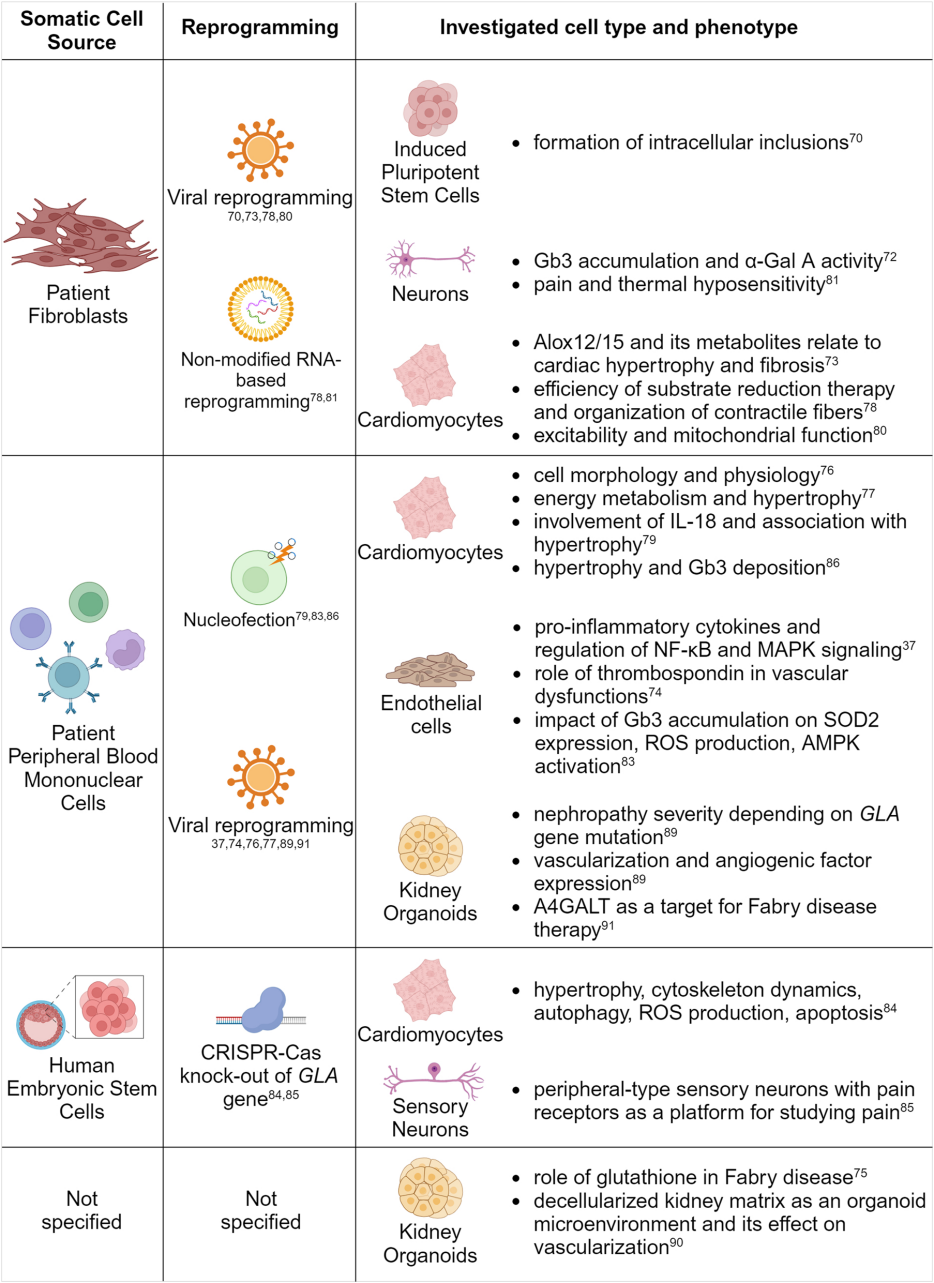
Induced pluripotent stem cell models (2D and 3D) for Fabry disease

The first human Fabry iPSCs were generated from dermal fibroblasts using a Sendai virus. These iPSCs indeed showed an accumulation of Gb3 [70], but could not be differentiated into mature cell types. This is no longer an obstacle, as a multiplicity of iPSCs derived from Fabry patients’ somatic cells have ever since been generated [71] and successfully differentiated to obtain various types of cells from different tissues [37, 72–75], making it possible to switch the research focus to more specific concerns. A summary of the stem cell models available for Fabry disease is depicted on Fig. 4.

It is of particular interest to derive cells from organs that are mostly involved with either disease complications or premature death. Since heart failure is one of the leading causes of death in Fabry disease, there has been considerable effort to establish disease models based on iPSC-derived cardiomyocytes (iPSC-CMs). In three concurrent studies, patient-specific iPSCs were derived from peripheral blood mononuclear cells and subsequently differentiated into iPSC-CMs [76–78]. In one of these studies, the patient was female [76] and the authors were able to generate an isogenic control line in parallel to the diseased iPSC line, since X chromosomes are known to undergo random X-inactivation and, hence, monoclonal iPSC lines can be derived from somatic cells with an active healthy or diseased X-chromosome. The authors observed Gb3 accumulation in diseased iPSC-CMs, but not in isogenic control cells. However, they were unable to show cellular hypertrophy and rather observed a cardiomyocyte shrinkage. In contrast, Chou et al*.* indeed observed a cellular hypertrophy [77], in addition to evaluating the energy metabolism of diseased iPSC-CMs. In the third study, where iPSCs were generated from PBMCs of 12 different Fabry patients carrying the same mutation in the *GLA* gene and used to generate cardiomyocytes, reduced enzyme activity, increased Gb3 accumulation and hypertrophy could be observed in comparison to healthy donor-derived iPSC-CMs [79]. Microarray analysis in these cardiomyocytes revealed that interleukin-18 (IL-18) and its receptor were upregulated and are associated with cardiomyocyte hypertrophy and poor response to enzyme replacement therapy [79] and that combining a neutralizing antibody along with ERT potentiated the treatment efficacy. The same study also suggested that IL-18 should be used as a biomarker for left ventricular hypertrophy in Fabry patients.

In addition to peripheral blood mononuclear cells, also skin fibroblasts were used to derive iPSCs from Fabry patients and to differentiate these into CMs [73, 78, 80]. Itier et al. used this model to mimic the disease phenotype by showing Gb3 accumulation and demonstrating a Gb3 reduction in response to an in vitro applied substrate reduction therapy [78]. Using fibroblasts with the same mutation, Birket et al*.* derived iPSC-CMs that neither showed shrinkage nor hypertrophy [80], but an increase in excitability. In this same study, the authors could point out that the most overrepresented protein in the secretome of such cells was cathepsin F and that this was driven by the accumulation of not Gb3, but of LIMP-2, a lysosomal protein with suggested role in heart disease [80]. Moreover, there was first evidence that Fabry cardiomyocytes may have a lower mitochondrial membrane potential, which could point to a mitochondrial functional deficiency [80]. In the study of Chien et al., an increase of fibrotic markers, along with hypertrophy was observed and Alox12/15 was identified to be involved in the pathophysiology of the disease, particularly in the later stages of disease progression [73]. The in vitro derived iPSC-CMs displayed many characteristics that are also observed in affected patients, such as disorganized contractile fibers contained in periphery of the cells [78], altered electrophysiology, including increased excitability and altered calcium handling [80], cardiomyocyte hypertrophy and fibrotic markers[73]. This highlights the value of iPSC-based disease models for the investigation of patient-specific disease mechanisms.

Skin fibroblasts were also used to model the neuronal phenotype of Fabry disease. By using a RNA reprogramming method, skin fibroblasts were transformed into iPSCs and subsequently differentiated into neural stem cells and neurons [72]. However, unlike the patient fibroblasts from which they were first derived, such cells lacked Gb3 accumulation. In a similar approach, small fibre neuropathy has been modeled using sensory neurons differentiated from patient-derived iPSCs. In addition to showing persistent Gb3 accumulation, the authors could also demonstrate that these neurons presented a higher activity when stimulated with heat, which is a common trigger for neuropathic pain [81].

In the range of cells affected by the mutation in the *GLA* gene and contributing to the Fabry disease phenotype, endothelial cells also represent an important part. Fabry patients have been shown to have an impaired endothelial function and affected vasoregenerative response [82]. Hence, several iPSC models for vasculopathy have also been described [37, 74, 83]. In one of these studies, thrombospondin 1 was identified to contribute to the dysfunction of vascular endothelial cells derived from four different Fabry patients each carrying a different mutation [74]. In another study, the increased expression of pro-inflammatory markers in presence of a *GLA* gene mutation was detected [37]. Upon correction of the mutation, iPSC-derived cardiovascular endothelial cells showed a decrease in cytokine secretion. The same study also showed that the mutation has an impact on NF-κB and MAPK signaling pathways. A third study showed that the production of reactive oxygen species (ROS) was increased in Fabry iPSC-derived endothelial cells along with a downregulation of the mitochondrial antioxidant SOD2 and an enhanced activity of the AMPK [83]. Treating HUVECs with Gb3, the authors demonstrated that Gb3 was responsible for suppressed SOD2 expression, increased ROS production and enhanced AMPK activation, finally resulting in endothelial dysfunction.

Disease models can also be created from genetic engineering, such as the use of the CRISPR/Cas9 system to induce mutations or a gene knockout and therefore express the phenotype of the disease. Embryonic stem cells have been altered to carry a *GLA* knockout and, upon differentiation into cardiomyocytes, showed increased cellular size, along with the dysregulation of proteins involved in cytoskeleton dynamics, affecting the exosome biogenesis and processes coupled to it, such as autophagy, which increases mitochondrial ROS production and increases cell death [84]. *GLA* knockout embryonic stem cells have also been used to model peripheral neuropathy. Differentiated cells showed markers for peripheral neurons, sensory neurons and pain channels and maintained these phenotypes for up to 40 days [85]. The authors concluded that this model would allow studying nociceptor properties in vitro.

Genome engineering with CRISPR/Cas9 has not only been used to introduce a knockout of the *GLA* gene, but also to correct gene mutations in patient-derived iPSCs [37, 74, 80, 86]. In an attempt to optimize gene correction efficiency, Chien et al. established a DNA transfection system based on 1,6-hexanedithiol (HDT)-conjugated maleimide-functionalized polyurethane grafted with small molecular weight polyethylenimine (PU-PEI600-Mal) [86]. Using HDT-conjugated PU-PEI600-Mal, an efficient delivery of sgRNA/Cas9 and homology-directed repair DNA template into patient-derived iPSCs was achieved. Targeted cells were subsequently differentiated into CMs, and these cells indeed showed a rescue of the disease phenotype including the absence of Gb3 accumulation and hypertrophy.

In general, 2D models also have their limitations. The non-natural environment of cells that grow in a monolayer, while attached to an artificial matrix is a major difference between the native tissue and such cultures, together with the lack of microenvironment provided by the actual tissue architecture [87]. Therefore, models that more reliably resemble the in vivo situation and that are reproducible and effective are needed.

### Three-dimensional Fabry disease models

A promising model for finding new therapies and personalized medicine is the 3D cell culture model. More complex than the 2D, it resembles a tissue better than a monolayer culture and it promises to bridge the in vitro and the in vivo studies. One option of 3D cultures is the organoid. There are different definitions for an organoid. For some authors, an organoid must contain more than one cell type of the modelled tissue, it should fulfil some functions of the resembled tissue and the organization of the cells should be similar to the actual organ [88]. Other authors define organoids as a 3D structure containing only one type of cell and, those structures with more cell types, are considered as engineered tissue [87]. However, even if several authors define the terms differently, there is a consensus in the literature that 3D cultures are more translational and resemble a tissue better than 2D cell cultures. This is especially important for disease modelling and the translation of its findings to patients.

The use of organoids in disease modelling is just starting. For Fabry disease, not many options for 3D cultures are available yet, but they do exist. Kidney organoids have been produced from iPSCs derived from Fabry patients and showed a typical segmented tubular structure and they showed the two most typical features of Fabry disease – decrease in α-Gal A activity, along with Gb3 accumulation [89]. The same study was also capable of showing a different severity of the phenotype depending on the type of mutation the cells harbored and that these organoids could represent Fabry disease nephropathy. The Fabry organoids showed accumulation of lipids inside the cells, which decreased upon enzyme replacement treatment. Compared to the healthy organoids, the vascularization in Fabry organoids was impaired, along with a decreased expression of angiogenic factors, which is compatible with the disease phenotype. The vascularization, importantly, could be rescued by ERT.

In an attempt to increase vascularization and maturation of human kidney organoids, another study extracted a decellularized extracellular matrix (ECM) to process it into a hydrogel and use it to create a better microenvironment [90]. This strategy revealed that the healthy organoids not only interacted with the matrix, but also remodeled it and this promoted a better vascularization when compared to organoids cultivated with Matrigel. In the future, ECM technologies could also be applied to other types of organoids to promote their maturation.

In a recent study, the organoid technology was combined with a novel substrate reduction approach. Cui et al*.* achieved a substrate reduction by knocking out the gene *A4GALT*, responsible for coding two enzymes involved in the biosynthesis of Gb3, in iPSC-derived kidney organoids [91]. This approach resulted not only in the lack of Gb3 build up, but also in the decreased expression of several genes involved in the progression of Fabry disease, like genes involved in cell death and angiogenesis. Results also suggested that a double knockout model of *GLA* and *A4GALT* was more similar to a wild type than to its *GLA* knockout parental cell line. Although this approach showed promising ideas, the authors pointed out that kidney organoids in general resembled a rather immature form of the kidney and the Gb3 deposition with subsequent tissue damage in the patient takes a much longer time than the 3-week cultivation of these in vitro models.

Patients with Fabry disease also show a significant imbalance in oxidative stress status, even in phenotypes where organ damage has not been identified yet [92]. Following this line of research, kidney organoids have also been used for transcriptomic analysis, which revealed a possible novel strategy for the treatment of the disease [75]. Glutathione is an endogenous low-molecular-weight compound that plays an important role in cellular redox and protects the cell from reactive oxygen species and the kidneys are dependent on a proper supply of it to maintain their functionality. The authors could identify a decrease in the metabolism of glutathione and that, upon restoring the normal levels in the organoids, the oxidative stress reduced significantly, and cellular damage improved. Targeting glutathione metabolism and oxidative stress levels could be yet another approach to developing a new treatment.

Although 3D models have a bright future, there are currently certain limitations that need to be addressed. Great effort has been made to try and improve certain aspects of 3D models, such as application of microfluidic devices or organ-on-a-chip that provide microchambers and channels that improve nutrient distribution and growth factors [93]. No available 3D model thus far has the complete physiology regarding maturation, cell composition and functions in the same way that they would have in their native tissue [94]. Another limitation is the lack of vascularization in the current models, as this is a needed feature for an exact reproduction of the native tissue in regard to nutrient accessibility and clearance of dead cells [87, 94].

### Limitations and perspective of the current in vitro Fabry models

To summarize the currently available models mentioned in this review, Table 1 provides a comprehensive overview of the different models and their key features including *GLA* genotype, Gb3 phenotype and treatment modality tested.

**Table 1:** Overview of in vitro models of Fabry disease

Mutation	α-galactosidase A activity	Gb3 accumulation	Treatment response	Reference
Q279E	< 2 nmol/mg/h	Yes (patient fibroblast)	DGJ and galactostatin bisulfide increased α-Gal A activity	[26]
Unknown	Not measured	Yes (patient fibroblast)	Not tested	[38]
Unknown	Not measured	Yes	Clearance of Gb3 deposits upon ERT and influences in pain associated membrane channel	[39]
p.Q280K, p.R342Q, c.801ins36, c.delEx2	< 0.1 nmol/mg/h	Yes	Clearance of Gb3 deposits upon ERT	[40]
Deletion on exon 3 and 4 on patient cell line, testes plasmids with p.C56Y, p.L300F, p.D244H, p.V269M, p.Q280K, p.A230Tand p.E341D	< 1 nmol/mg/h	Yes	Acetylsalicylic acid raises ﻿α-Gal A activity together with DGJ in amenable mutations	[42]
Unknown	< 12 nmol/mg/h	Yes (patient fibroblast)	Gene therapy reduced up to 90% of gb3 deposits	[44]
Unknown	Not measured	Induced accumulation	Not tested	[45]
Unknown	Not measured	Induced accumulation	Not tested	[46]
Unknown	Not measured	Induced accumulation	Pentosan polysulfate reduced production of IL-1β and TNF-α	[47]
c.159C > G p.(Asn53Lys), c.400 T > C p.(Tyr134His), c.680G > C (p.Arg227Pro), c.815A > T p.(Asn272Ile), c.907A > T p.(Ile303Phe) and c.1163_1165delTCC (p.Leu388del)	0—53 nmol/mg/h	Yes	Up to 67% rescue with DGJ chaperone	[49]
R112H	< 20 nmol/mg/h	Yes	Reduction of lysosomal deposits with 24 h of recombinant ﻿α-Gal A enzyme	[50]
c.109G > A (p.Ala37Thr, A37T), c.730G > C (p.Asp244His, D244H), c.898C > T (p.Leu300Phe, L300F), c.838C > A (p.Gln280Lys, Q280K), and c.805G > A (p.Val269Met, V269M)	< 15 nmol/mg/h	Yes	Curcumin treatment increased ﻿α-Gal A activity from 1,4 to 2,twofold	[51]
p.N215S and p.L294S	< 11% of wild type levels	Yes	Increase ﻿α-Gal A activity and reduction of Gb3 deposits for p.N215S, but not for p.L294S using DGJ	[52]
c.280 T > C, p.Cys94Arg	40% of wild type levels	Yes	Not tested	[53]
M296I, N263S, G360D, R301Q, D165V, A288D and R100T	0%, 5% and 15% of wild type levels	Not checked	Not tested	[54]
Frame shift from exon 1	0% of wild type (HEK model)	Yes (patient fibroblast)	Proteasome inhibitor increased rh﻿α-Gal A by twofold and 30% increase of Gb3 clearance in patient fibroblasts	[55]
c.898C > T p.L300F; c.838C > A, p.Q280K; c.730G > C, p.D244H; c.902G > C, p.R301P	10% of wild type in L300F	Not mentioned	DGJ helps to stabilize enzyme in different pHs	[56]
None	Not checked	Induced accumulation	High concentrations of DGL to inhibit function	[57]
Not specified	Not checked	Yes (patient cells)	ERT reduced Gb3 accumulation in patient cells	[58]
None	70–80% reduction upon cloroquine treatment	Yes	Not tested	[59]
Disruption of exon 1	Not detectable on knock down or knock-out model	Not mentioned	α-Gal A and eliglustat did not significantly decreased vWF	[60]
Unknown	Less than 2% of wild type	Yes (lyso-gb3)	Improvement of glycocalyx thickness upon ERT or migalastat	[61]
None, GLA shRNA knockdown	45% and 15% of control	Yes	Not tested	[63]
None, GLA shRNA knockdown	Around 4% of control	Yes	Clearance of Gb3 deposits upon ERT combined with DGJ, but not reversal of other signaling disturbances	[64]
Insertion/deletion on exon 1	Less than 2% of wild type	Yes	ERT reverses Gb3 deposits, but not cellular injury	[65]
None, GLA siRNA knockdown	Not directly measured	Not mentioned	Not tested	[66]
None, gla shRNA knockdown	< 30% of control	Yes	Not tested	[67]
W162X	Not measured	Yes	Not tested	[70]
c.959A > T	Not measured	Yes	Not tested	[71]
IVS4 + 919G > A	< 20 µg/mg/h (cardiomyocytes)	Yes	Early α-Gal A administration reduced cardiomyocyte hypertrophy	[72]
c.803_806del, p.L268fs*1; c.658C > T, p.Arg220*; c.334C > T, p.Arg112Cys; c.1045 T > C, p.Trp349Arg	< 1 µmol/mg/h	Yes	aberrant angiogenic function even upon α-Gal A	[73]
Corrected IVS4 + 919G > A	Restored to wild type levels	No	Gene correction led to functional enzyme	[74]
Deletion on exon 1	Not measured	Yes	Glutathione treatment reduced oxidative stress in Fabry kidney organoids, ERT does not fully reverse damage	[75]
c.779G > C	< 5 nmol/mg/h on mutant and > 15 on normal clone	Yes	Not tested	[76]
IVS4 + 919G > A	< 10 nmol/mg/h	Yes	ERT led to Gb3 clearance but did not rescue energy metabolism	[77]
c.485G > A, W162X; c.658C > T, R220X	Not detectable	Yes	Treatment with glucosylceramide synthase prevent accumulation and cleared gb3 deposits	[78]
IVS4 + 919G > A	< 1 µmol/µg/h	Yes	Lower lyso-Gb3 levels and reduction in cardiomyocyte size, potentiated by IL-18 neutralizing antibody	[79]
c.458G > A and c.658C > T	< 1% of wild type	Yes	Not tested	[80]
c1096C > T, c.568delG, c.708G > C	< 0.6 nmol/mg/h in all lines	Yes	Gb3 deposits were cleaved up to 27% upon 24h α-Gal A treatment	[81]
Exon 1 deletion	Not detectable	Yes	Not tested	[85]
IVS4 + 919G > A	< 10 nmol/mg/h	Yes	Non-viral gene correction	[86]
c.969delC, p.Leu324Trpfs ∗ 24; c.263A > G, p.Tyr88Cys	4,6 × 10^3 and 4,9 × 10^4 pmol/mg/h	Yes	not tested	[89]
c.901C > T, p.Arg301X*	Knock out: 3,9 × 10^3; GLA and A4GALT-KO: 1,6 × 10^4; fabry patient 6,2 × 10^3; fabry patient with A4GALT lnock out: 5,9 × 10^3	Yes (fabry patient line and knock-out)	Suppression of *A4GALT* decreased Gb3 accumulation	[91]

Despite several advantages and flexibility offered by these models, there remain major obstacles. Often, 3D models are more immature than the native tissue and are simplified versions, meaning that they sometimes lack tissue specific cell types like resident immune cells [94]. Another shortcoming is the lack of vascularization, which restricts the exchange of oxygen and nutrients, interfering also with the removal of metabolic waste [95]. This limitation often means a limited size of the microtissues and, in turn, narrows its functionality. Nevertheless, 3D models represent valid advantages concerning the translatability into human disease model. Animal models can provide us with cues to developmental, physiological and pathophysiological cues, but they are often expensive, ethical concerned laden and do not effectively show human specific responses to diseases or treatments [96]. Alternatively, the development of perfusable cultures are a major advance in this matter.

In cardiac 3D models, iPSC-derived cardiomyocytes present a more fetal-like metabolism, especially concerning the electrophysiological activity [97] and the use of isolated human or other animal cardiomyocytes provide not only limited availability in numbers, but also in integration with other cells included in the models [96]. Other organs are more readily replicated, even when presenting complex structures. Fabry kidney organoids present important functions of the kidney, showing glomerular and tubular-like structures, as well as differences between healthy and diseased [75]. Other limitations are being currently addressed, such as the interaction between organs, by organ-on-chip approaches.

### Outlook

The generation of new models that resemble the disease and the identification of new treatment approaches are on the rise in Fabry research. The next steps should focus not only on finding new pathways to reduce the burden of the symptoms in patients, but on trying to model various tissues as well. Clinical trials for testing new drugs are currently ongoing, such as a new GCS inhibitor with limited off target activity (NCT06114329), as well as testing for new types of treatments involving stem cell transplant with CD34 positive cells transfected with lentivirus to produce α-galactosidase A (NCT02800070) [30], or new drugs to combine with traditional treatments, like dapaglifozin for patients with cardiac and renal involvement (NCT05710367). Other studies are focusing on reinforcing the safety and efficacy of drugs already available Fabagal^®^ and comparing to its active comparator (NCT06081062), or combined enzyme replacement therapies with Fabryzyme^®^ and agalsidase beta (NCT05843916), and pegunigalsidase alfa to assess safety, pharmacodynamics and efficacy (NCT06328608), as well as gene editing studies involving AAV vectors to try more permanent approaches of treatment (ChiCTR2300073886, NCT05629559, NCT04040049). Gene therapy with AAV is being tested as a treatment for Fabry disease for over 20 years [98, 99] and shows that one injection could lead to expression of α-galactosidase A, correcting the phenotype persistently. More recently, studies have used optimized AAV vectors, obtaining higher tissue and plasma transgene expression, complemented by normalization of Gb3 storage in plasma, kidney and heart [100–102], which provides the basis for advancing the research towards trials with patients, in particular patients with nonsense or missense mutations. In vitro models can also be useful for finding promising new avenues of treatment or providing preliminary data for such studies on human specific tissues. Models from patients and healthy subjects can also help. Several organoids have been generated for healthy tissues, which could, in the future, be adapted to Fabry disease. The modeling of cerebral organoids in Fabry disease would be of relevance and models of fused brain organoids have already been developed for healthy cells [103]. The heart is also important in Fabry disease. The modelling of diseased heart organoids and cardiac tissues would be of great importance on the research of phenotype progression and treatment in Fabry disease. There are many different types of models for investigating heart diseases and physiology, which include spheroids, organoids, engineered cardiac microtissues, bioprinting and even the use of ex vivo models [97]. Models have been generated for myocardial infarction and drug toxicity [104], cardiac organoids using a multicellular approach [105] and even the generation of iPSC-derived myocardium [106]. Multi-organoids on a chip have been developed to hold up to six different microtissue types to study their interactions and offers a more complete platform for drugs screening [107] and demonstrating the overall potential of these models.

The use of in vitro models will continue to grow in the next years. As of the beginning of 2023, the U.S. Food and Drug Administration no longer requires that drugs are tested in animal models if they have been tested in cell-based assays, organ chips or microphysiological systems. This is a major milestone not only for animal welfare, but also for translational research, as these systems are derived from human tissues and do not have the species-specific metabolic differences of many available animal models.
